# The effect of vocational rehabilitation on the use of health care services in Finland

**DOI:** 10.1093/eurpub/ckac131.292

**Published:** 2022-10-25

**Authors:** H Rinne

**Affiliations:** Social Insurance Institution of Finland, Helsinki, Finland

## Abstract

**Background:**

Because vocational rehabilitation is a separate service from other health services, there may be interruptions at their interface. We examined whether the use of health services changes before and after rehabilitation.

**Methods:**

Longitudinal individual-level register-based data was utilized on all individuals aged 15-60 living in the city of Oulu, Finland and starting vocational rehabilitation in 2014-2015 (N = 792). We compared their use of outpatient health care services in 3-month periods from 1.5 years before to 1.5 after the rehabilitation period to the total population of the same age and to the propensity score matched controls. Several socio-demographic factors and sickness and employment histories were used for matching.

**Results:**

According to the preliminary results, rehabilitees had on average 1.5 visits to outpatient health care services in the 6th quarter before the start of rehabilitation, twice that of the total population. In the 4th quarter before the start of rehabilitation, the number increased to 1.8. After the rehabilitation period, the quarterly number of visits were at the same level as in the beginning of the follow-up. The biggest changes took place in the use of occupational health services. Changes were modest in public health care services. In other services changes were minimal. Compared to the propensity score matched controls, vocational rehabilitation did not appear to have an effect on the use of health care services.

**Conclusions:**

The use of health care services is more common before vocational rehabilitation than after it. The effect of rehabilitation on the use of health care needs further analysis.

**Key messages:**

• The pattern of the use of health care services changes in the course of vocational rehabilitation. The changes are mainly due to visits in occupational health care services.

• The use of health care services is most common before rehabilitation. However, vocational rehabilitation does not appear to have an effect on the use of outpatient and inpatient health care services.

